# Introduction: Invasive Species, Global Health, and Colonial Legacies

**DOI:** 10.1093/jhmas/jrae042

**Published:** 2024-12-09

**Authors:** Jules Skotnes-Brown, Christos Lynteris

**Affiliations:** University of St Andrews, Scotland, UK; University of St Andrews, Scotland, UK

**Keywords:** invasive species, colonialism, medicine, health, disease, environment, xenophobia

## Abstract

Bringing together seven papers spanning Southern and Eastern Africa, North America, England, and India, this special issue explores the historically neglected connections between invasive species and health in the long twentieth century. Drawing upon perspectives from medical history, the history of science, environmental history, and environmental as well as medical anthropology, the papers analyze the entanglements of invasive species and zoonotic disease, food security, pesticide, crime, and ecosystem health. This introduction provides an overview of the historiography of invasive species and argues the importance of studying the historical connections between invasives and health. It also historicizes the relations between animal invasions, technoscience, power, and colonialism.

On 28 July 2023, the French center-left daily *Libération* carried a menacing image of a mosquito on its front cover, showing a striped insect as if seen through a magnifying glass. The title in red and black capital letters read: “TIGER MOSQUITO. AGAINST THE INVADER.”^1^ The lead article explained that, a “product of globalization,” the tiger mosquito, “humanity’s greatest enemy,” had been found in France since 2004 but was now posing new risks as climate change was enabling the spread of dengue fever by this insect.^2^ Indeed, over the summer of 2023, alarm over dengue-fever-spreading tiger mosquitos resonated across French media. *Le Monde*, at the political antipodes of *Libération*, carried a video-article titled “How the tiger-mosquito colonised France,” explaining how this “conquest” took place.^3^ In this case, rather than globalization, the means of the invasion were identified as rubber tires from Southeast Asia, where mosquitos supposedly find refuge and an ideal reproductive habitus. The video used maps of main road arteries in France (arguing that the mosquitoes travel in cars and parcels), showing a time-lapse of the geographic propagation of these “super-mosquitoes” as well as of projected temperature rises in Europe over 2030-2050, and maps of dengue, Zika, and chikungunya epicenters in France. These multi-species, epidemiologically-inspired visuals unmistakably echoed maps of military invasion. From the Left, with its language of globalization, to the Right, with its language and visual tropes of conquest and colonization (a term recuperated by the extreme-right to target Arab and Muslim communities in France), the disease-spreading mosquito appears to form a common discursive and material ground for developing political agendas that find common cause in the defense of a motherland imagined to be under attack by foreign agents carrying foreign diseases.

These fears of mosquito “invasions” into Europe are revealing of longer processes of politicizing invasive species as a serious risk to western public health, going back to imperial panics over diseases border-crossing into the West from Africa or Asia.^4^ Yet in reality, the brunt of recent health problems caused by invasive species has largely been borne by countries in the Global South. In the midst of the first coronavirus wave in China, the worst locust swarms of seventy years were ravaging East Africa. On 29 January 2020, the Food and Agriculture Organization of the United Nations warned that rapidly multiplying desert locusts presented an “unprecedented threat to food security and livelihoods in the Horn of Africa.”^5^ The organization wrote that the swarming insects could “eat the same amount of food in one day as 35,000 people,” had already “invaded 13 countries,” and predicted that South Sudan and Uganda were at risk.^6^ A month later, this prediction proved correct, and the Ugandan government deployed veteran soldier Major General Samuel Kavuma as a “locust commander” to wage “war” against this “formidable enemy.”^7^ By March 2020, Kavuma reported that his forces had “managed to control the locust invasion.”^8^ Other countries were not as lucky. Mwende Kimanzi, a Kenyan farmer, reported that the insects had almost completely destroyed her crops and left her family with hunger.^9^ The Ugandan *Daily Monitor* blamed a period of “long dry weather followed by a series of cyclones that dumped water in the East Africa region” for creating “excessively ideal conditions” for locusts to breed.^10^ According to entomologist David Hughes, these unusual weather patterns were a direct result of climate change.^11^

In an era of such anthropogenic disasters, many scientists now argue that species invasions are a global health problem through their role in spreading infectious diseases, devouring food supplies, or undermining ecological stability.^12^ Yet as these vignettes reveal, fears of invasive species are often shaped by prejudices with long roots in colonial discourse and a distinctly modern faith that technoscience and military might can repel and eradicate them and the diseases or health problems they carry or cause. This special issue brings together studies that examine the entanglement of medical, environmental, and animal histories of species invasiveness. In so doing, it aims to go beyond traditional disciplinary oppositions between approaches focused on the political ecology of species invasiveness and the political discourse around them. The articles in the collection ask how invasive species and their framing by diverse technoscientific regimes have shaped disease ecologies, how they have affected food security and wellbeing, how they have been scapegoated for human-made disasters, and how they have contributed to the development of new environmental and global health frameworks, regimes, and practices.

## Invasive Species: Multispecies Histories and Power Relations

Although environmental historians have long been interested in invasive species, dating at least as far back as Alfred Crosby’s famous books *The Columbian Exchange* (1972) and *Ecological Imperialism* (1986), which stressed how the colonization of the Americas resulted in the proliferation of invasive biota, critical historical and theoretical problematizations of the invasive species concept itself only became prominent in the 1990s and 2000s.^13^ Indeed, it is now generally accepted that, “one of the defining features of European imperialism was the purposeful introduction of foreign organisms, especially plants and animals, into every country and colony in the world.”^14^ Growing political and environmental concern over the impact of exotic species on the degradation of the environment has since led humanities and social science scholars to critically examine the concept of invasive species and its histories. Many early theoretical works critiqued the association of local species with nationalism and hatred of those foreign, and problematized the distinctions between native and exotic species, with “a coherent [biological] theory of invasion [remaining] elusive.”^15^ As Stefan Helmreich has shown in his examination of scientist taxonomies of invasive and native species in Hawai’i, this elusiveness in the definition of species invasion or invasiveness has been epistemically, politically, and socially productive in that it forms the ground for social negotiation of responsibility over environmental change, as well as for claims to more-than-human agency.^16^

While some scholars have criticized legal, cultural, and scientific indictments of invasive species as militaristic, anthropocentric, and outright xenophobic, others have insisted that there is value to an invasive species framework in protecting local biodiversity.^17^ Across this spectrum, a key contribution to this conversation has been to historicize invasiveness: what makes a species “invasive,” in other words, is shaped by relations of power in societies. And in turn, “a sizable body of research shows how examples of efforts to control introduced and invasive species often reinforced unequal gender, power, and race relations.”^18^ Historians, anthropologists, and geographers have explored how various governments, scientists, and social elites came to frame and act upon invasive species according to situated economic, political, and biopolitical interests and agendas. In different contexts across the globe, framing and mastering invasive species has formed an important part of colonial, anticolonial, nationalist, and socialist governmentalities. More often than not, such framings and policies are negotiated and contested by communities affected by them.^19^ To give but one historiographically prominent example, in his study of the prickly pear in the nineteenth-century Cape Colony, Lance van Sittert argued that biological invasions were “historical processes primarily shaped not by the biology of the invader, but the shifting cultural values of the invaded society.”^20^ Van Sittert showed how the prickly pear’s status as invasive was shaped by colonial class and race relations, and how the plant was blamed for broader social ills. In the eyes of the British political establishment, this plant was responsible for everything from alcoholism and idleness to the poisoning of cattle. The Cape bourgeoisie blamed Black people and poor Whites for spreading this cactus uncontrollably and demanded its eradication. This, however, provoked resistance from poorer farmers who used it as food, fodder, or sold its parts for income. In this case, the status of an invasive species as a negative force on Cape society was a matter of power relations between elites and underclasses.^21^

If what counts as invasive is shaped by structural relations of power between human groups, xenophobic discourse has played a key role in the formulation of “ready-made scripts of invasive species,” and strategies for their control.^22^ In his cultural and social history of invasive species in the United States, Peter Coates has argued that American obsessions over “alien” flora and fauna and immigrant humans were discursively and biopolitically connected. Coates demonstrates that American perceptions of foreign plants and animals were shaped by the same xenophobic attitudes towards humans that pervaded nineteenth- and twentieth-century American society.^23^ This approach has been recently further developed by Jeannie Natsuko Shinozuka. Homing in specifically on Asian plants, insects, and people in the western US, Shinozuka’s *Biotic Borders* shows the entanglements of concerns over foreign species and anti-Asian racism.^24^ Racial prejudices and legislation against Asian Americans, she argues, have more-than-human roots. Even the notorious Chinese Exclusion Act of 1882 was part of a multispecies fixation with biological nativism and hatred of foreign biota. Along with “concerns over national and racial purity,” she argues, “US officers viewed a truly healthy body as one insulated from its larger ecological context.”^25^

While humanities and social science works on invasive species have always included multispecies elements, Shinozuka’s writing points to an important turn in the scholarship on species invasiveness in the humanities and the social sciences away from primarily human-oriented approaches to more-than-human ones. This turn, which centers animals and plants, often alongside human actors, tackles questions of species invasiveness from the prism of the Anthropocene, challenging nature/culture dichotomies through nuanced understandings of invasive ecologies.^26^ Examining approaches to invasive species as “the principal threat to biodiversity on islands in Mexico,” Emily Wanderer has stressed how some species become naturalized as “inherently problematic, erasing the ways in which invasive species only become destructive when they move or are moved into new ecological niches.”^27^ However, it is by focusing away from the militaristic discourse of anti-invasive campaigns and policies, and by paying “closer attention to how species meet,” argues Wanderer, that we can begin to understand both the complex agencies of species designated as invasive and how they and scientists and non-human animals actually interact on the ground.^28^

Paying attention to multispecies relations does not mean losing sight of the political and economic conditions of species invasiveness. As Laura Ogden has shown in her important work on invasiveness and loss in Tierra del Fuego, multispecies approaches are indeed vital for enriching our understanding of ecological imperialism as a global process comprising in “not just the remaking of landscapes to look like Europe but also [...] of remaking nonhuman life through the constitution of new multispecies assemblages.”^29^ These studies have contributed much to our understandings of both invasive species and to notions of historical agency. However, more-than-human approaches in colonial contexts must take care not to reproduce colonial discourses. In his rich analysis of the introduction of the Indian mongoose into Jamaica, Matthew Holmes has shown how multispecies approaches are “indispensable” to environmental and colonial history, but can also pose conceptual difficulties.^30^ The mongoose, which was introduced to control rats on the island, quickly became a framed as a problem species, proving how “ecological reality could be interpreted to harmonize with the wider imperial project.”^31^ Colonial celebrations of an agentive “balance of nature” which supposedly brought the mongoose under control, served to “rationalize British acclimatization in Jamaica,” stymie “criticism of plantation agriculture,” and mask the human-made nature of this ecological disaster.^32^

The story of invasive species management, however, is not merely a tale of complete colonial power over the environment. In many ways, the “control” of invasive species was sheer fantasy, which exposed the inability of governments, international organizations, public health authorities, and conservation groups to manage the environment. Jackson Perry’s analysis of European sparrows in Algeria, for example, shows how the “violent defense of settler economic power […] became an inter-species conflict” between birds and humans.^33^ These birds challenged colonial power over the environment and the dominion of nature in the region. In an 1879 article, resembling the French newspaper vignette with which this introduction began, one author claimed that “the French have conquered the Arabs and won their colony, only in their turn to be conquered by the sparrows.”^34^ Writing on locusts in 1920s South Africa, Irish South African naturalist Frederick FitzSimons similarly argued that insects were “the real lords and masters of the world” and that colonial attacks did “about as much damage to the insect enemy as shooting into a flight of migratory locusts with a single rifle.”^35^ In this same context, fears of invasive species could also be mobilized for subversive reasons. In 1934, Black South African journalist, writer, and editor Rolfes Robert Reginald Dhlomo used the imagery of locusts to circulate revolutionary rhetoric in South Africa.^36^ Similarly in Australia, Haripriya Rangan and Christian Kull have demonstrated how invasive species in the Outback challenged racist national histories and mythologies, revealing the interconnections of Australia and the Indian Ocean world.^37^

## Invasive Species, Medicine, and Health

In spite of the extensive corpus of works on invasive species in the humanities and the social sciences, little attention has been paid to the relation between invasive species and medical or health-related questions. Whereas in the social sciences questions of species invasiveness and questions of disease and health have formed the subject of joint examination under the rubric of biosecurity studies, with the notable exception of the introduction of infectious diseases that resulted from the “Columbian Exchange,” these questions are usually siloed within environmental and medical subdisciplines, respectively.^38^ Recently, significant attempts have been made to bridge these subfields. To name one example, Gabriel Lopes has analyzed the political, institutional, and public health implications of the introduction of the malaria-carrying African *Anopheles gambiae* mosquito into Brazil in the 1930s. This insect, although neglected by public health authorities in the 1930s, took on “unprecedented importance in the 1940s,” when it was denounced as “the worst immigrant that could reach us from the north,” worse even than “an invasion of heretics, Communists, or Nazis.”^39^ Nevertheless, the historiography of medical and public health concerns with species invasiveness remains largely unexplored, and it is common for historians of medicine to attribute scientific interest on species invasiveness as a driver of zoonotic infection to the impact of the work of Charles Elton on the emergence of disease ecology frameworks from the 1920s onwards.^40^ This attribution, based as it is on Global North-centered histories of medicine, overlooks three decades of medical problematization of species invasiveness, as this unfolded in the context of the third plague pandemic (1894-1959), a devastating global epidemic of bubonic plague that led to more than twelve million deaths and formed one of the most important springboards for modern epidemiology.^41^ On the one hand, the emergence of disease ecology (by K.F. Meyer in the US and Evgeny Nikanorovich Pavlovsky in the USSR) in the 1920s and 1930s, as a framework for understanding both sylvatic plague and other zoonotic and vector-borne diseases, was indeed transformative to forging together problematizations of infectious diseases and species invasiveness.^42^ On the other hand, for as long as thirty years preceding this ecological framework, it was a bringing together of colonial and racialized medical framings of plague with pre-existing concerns over the migratory patterns of rats that established the first global network of scientific debate and research on a non-human animal “invasion” as a major threat to global health, which eventually established rats as the first pandemic animal villains in modern human history.^43^

Bringing together scholars of the environmental and medical humanities, this special issue aims to elucidate the medical and health dimensions of species invasiveness, and to open up a space of scholarly discussion on how discourses on invasive species and practices for their control affect social and multispecies worlds in different historical contexts. How have various concepts and framings of species invasiveness as a medical/health problem shaped relationships between humans and non-humans? What forms of governance become fostered by the medicalization of species invasiveness? How does the entanglement of concerns and policies about invasive species and public health rely upon and reinforce discriminatory and racist framings of human life? And how, in turn, do ideas about species invasiveness unsettle hegemonic ideas about health? In which ways have the entanglements between invasive species and health been mobilized by subalterns or marginalized groups? And how can we account for multispecies affordances and agencies at the junction of medical and environmental histories of invasive species?

The articles in this special issue seek to address these issues across four continents from the late-nineteenth to the early-twenty-first century. Admire Mseba’s article on the transnational politics of locust control in twentieth-century Southern Africa shows how swarms of crop-devouring locusts sparked international collaboration between Southern African nations over their control. Trans-border attempts to control and monitor locust swarms were inaugurated in the colonial era, where colonial prejudices were grafted onto the politics of locust control in the British settler colonies of South Africa and Southern Rhodesia. Here, “outbreak areas” – tracts of country in which locust swarms formed – were correlated with areas in which Indigenous Africans lived and framed as risks to be managed through inter-colonial cooperation. After decolonization, many southern and east African countries unsurprisingly did not wish to cooperate with these racist regimes and formed a new international locust control organization, the IRLCO-CSA. However, cutting off the wealthier settler colonies meant a severe loss of funding, forcing the IRLCO-CSA to rely upon international donor funders. These funders supplied southern African nations with hazardous organocholorine chemicals, to detrimental ecological and human health effects, which eventually failed to control locusts as pesticide campaigns neglected the ecological, social, economic, and political forces that enabled these insects to flourish.

In their micro-global history of plague on the border of Namibia (then South West Africa) and Angola (1932-1937), Jules Skotnes-Brown and Matheus Alves Duarte da Silva likewise show how South African, Angolan, Portuguese, and South West African imperial authorities, along with Indigenous Oukwanyama political elites, collaborated in the study and control of plague. In the early days of the outbreak, the usual xenophobic and racist blame for the disease was passed around, resulting in the deterioration of political relations among different powers in the region. However, in what was both a scientific mission and a diplomatic exercise, scientist Louis Fourie shifted the blame away from humans and onto “migratory” gerbils that allegedly spread plague from its endemic area in the Northern Cape all the way to the Angolan border. Creating an animal “epidemic villain,” inaugurated a period of collaboration and cooperation between all human groups in the region over the study and control of gerbils.^44^ However, this blaming of rodents for the outbreak also had the effect of side-lining alternative theories about the important role played by colonial trade and labor practices in the spread of plague.

Indeed, in many cases shifting the blame for broader anthropogenic environmental and health problems onto animals has diverted attention away from the role played by extractive industries in deteriorating the environment and human health. This was not only prevalent in past colonial contexts but also remains common in the heart of the former British Empire. Maddy Pearson shows how American mink were blamed for broader environmental ailments in south England through a close analysis of the River Beane in the 2010s-2020s. This river, which was declared ecologically dead by activists in 2019, has become the subject of intensive environmental campaigning and ecological restoration efforts. Here, Pearson shows how activists framed American mink, a foreign species introduced for the fur trade industry, as detrimental to the chalk stream ecosystem in order to galvanize support for the restoration the river. Pearson shows how mink were reductively blamed for the decline of the local vole populations (a charismatic flagship species of rural south England), taken as symptomatic of poor ecological health, and made targets for complete eradication in service of restoring an imagined timeless, pre-imperial past to the river.

Likewise, in the US and Canada, Vincent Bijman’s paper shows how the construction of the sea lamprey as a “vampire from the deeps” (1940s-1970s) served to obscure broader environmental ills in the transborder Great Lakes region. This eel-like parasitic fish, which latches onto other fishes and feeds on their blood, was initially blamed for food security problems (the decline of fish in the region) as well as the loss of tourism funds from sport fishing. This drew attention away from ecological degradation in the region spurred by capitalist industries such as agriculture, logging, and mining. Fears of the impact of the sea lamprey were so great that they sparked unprecedented trans-border cooperation in the Great Lakes region, and justified flooding the water with a “lampricide,” even in the context of growing environmentalist opposition to chemical pesticides. However, in the 1960s and 1970s, the sea lamprey was reframed less as a disaster for food security and human wellbeing and more as an ecological problem to be managed for the sake of the health of the Great Lakes ecosystem.

Kenneth Reilly’s article address how invasive species became associated with social malaise and human underclasses, and thus were targeted by governmental biopolitics over humans, plants, and animals. Through his analysis of Japanese kudzu vine in late twentieth-century Atlanta, Reilly shows how the vine became entangled with broader social ills, including racism, crime, homelessness, drug use, and disease, leading to its ruling as a public health risk in 1988. Reilly’s analysis reveals how undesirable plants were correlated with human and animal underclasses – particularly unhoused people, snakes, and mosquitoes – but also shows how kudzu exposes the limits of capitalist and governmental power over the plant-life of Atlanta. In many cases, the plant was impossible to remove, or so cumbersome and difficult to do so, that eradication became a pipe dream. But the status of kudzu as a pestilent plant was contested. Marginalized human groups such as unhoused people depended on it for shelter, especially in the scorching summers. Thus, the control of kudzu was a facet of the exertion of capitalist power over a cityscape under the aegis of gentrification and economic growth.

Finally in his article on the “migratory rat” in British India, Christos Lynteris examines a case where species invasiveness does not concern national border-crossing but is imagined to take place within colonial urban environments. Lynteris shows how the figure of the migratory rat emerged within the first years of the third plague pandemic in India (1896-1899) as a way of explaining the spread of the disease within and between cities or other human settlements. Following studies and debates on what came to be know as “the rat’s progress” by both British and Indian doctors and public health officers, Lynteris argues that the “migratory rat” was not simply a convenient common ground for discussing the propagation of plague or laying blame for it. Instead it was a figure whose emergence marked a radical shift in epidemiological reasoning, as it came to replace another, hitherto hegemonic way of relating plague and rats, which had been crystalized since the 1870s in the figure of the “staggering rat.” Replacing the figure of the “staggering rat” by that of the “migratory rat,” Lynteris argued, “fostered a new, secularized idea of epidemic disease, as a process in and for itself, which unfolded in a linear, future-oriented temporality.”

Taken together, these articles break new empirical and theoretical ground through tracing historical lineages of the framing of invasive species as a problem for public health, food security, and the health of ecosystems on both local and global scales. In so doing, this special issue provides a critical historical and anthropological view on epidemiological and ecological fears of invasive species in the context of an ongoing pandemic and globally deteriorating environment.

## Footnotes

1
*Liberation*, 28 July 2023, cover image.

2 Nina Guérineau de Lamérie, “Moustique tigre en France: sus a l’envahisseur,” *Libération,* 28 July 2023, https://www.liberation.fr/environnement/biodiversite/moustique-tigre-en-france-sus-a-lenvahisseur-20230728_KHBVTNWGAZBBHBUDRW6TIC7NKY/.

3 Fanny Zarifi and Marceau Bretonnier, “Comment le moustique-tigre colonise la France,” *Le Monde*, 23 June 2023, https://www.lemonde.fr/planete/video/2023/06/23/comment-le-moustique-tigre-colonise-la-france_6178890_3244.html.

4 Jeannie Natsuko Shinozuka, *Biotic Borders: Transpacific Plant and Insect Migration and the Rise of Anti-Asian Racism in America, 1890-1950* (Chicago: University of Chicago Press, 2022). Shinozuka is also working on a book project that includes the Asian tiger mosquito as a global invasive species. See Shinozuka, “In the Eye of the Asian Tiger Mosquito: Insect Immigrants & Biocultural Racism in Italy,” conference presentation at the *American Society for Environmental History Annual Conference,* Boston, 22-26 March 2023. See also Alison Bashford, ed., *Medicine at the Border: Disease, Globalization and Security, 1850 to the Present* (London: Palgrave, 2004); Robert Peckham, ed., *Empires of Panic: Epidemics and Colonial Anxieties* (Hong Kong: Hong Kong University Press, 2015); Alison Bashford, *Quarantine: Local and Global Histories* (Basingstoke: Palgrave Macmillan, 2016); Alan Bewell, *Romanticism and Colonial Disease* (Baltimore: Johns Hopkins University Press, 1999); Gregg Mitman, “Ebola in a Stew of Fear,” *New England Journal of Medicine* 371 (2014): 1763–1765. For these fears in relation to emerging infectious diseases see Nicholas King, “The Scale Politics of Emerging Diseases,” *Osiris* 19 (2004): 62–76.

5 “FAO - News Article: Alarm over Desert Locusts Increases as New Generation of the Destructive Pests Starts Breeding in Horn of Africa,” *Food and Agriculture Organization of the United Nations*, 20 January 2020, https://www.fao.org/news/story/en/item/1258877/icode/.

6 Ibid.

7 Sally Hayden, “Uganda’s ‘Locust Commander’ Leads the Battle against a New Enemy,” *The Guardian*, 26 February 2020, https://www.theguardian.com/global-development/2020/feb/26/ugandas-locust-commander-leads-the-battle-against-a-new-enemy.

8 “Locust Invasion Has Been Contained – UPDF,” *Monitor*, 19 July 2020, https://www.monitor.co.ug/uganda/news/national/locust-invasion-has-been-contained-updf-1878960.

9 Reuters, “‘Nothing Left’: Kenyan Mother’s Fields Stripped by Locust Swarm,” *The Standard*, 2020, https://www.standardmedia.co.ke/eastern/article/2001361158/nothing-left-kenyan-mothers-fields-stripped-by-locust-swarm.

10 “Locust Invasion Has Been Contained – UPDF,” *Monitor*, 19 July 2020, https://www.monitor.co.ug/uganda/news/national/locust-invasion-has-been-contained-updf-1878960.

11 Interviewed in Rachel Nuwer, “As Locusts Swarmed East Africa, This Tech Helped Squash Them,” *New York Times*, 8 April 2021, https://www.nytimes.com/2021/04/08/science/locust-swarms-africa.html.

12 Peter Stoett, Helen E. Roy, and Aníbal Pauchard, “Invasive Alien Species and Planetary and Global Health Policy,” *Lancet Planetary Health* 3 (2019): e400–401.

13 Alfred W. Crosby, *The Columbian Exchange: Biological and Cultural Consequences of 1492* (Westport: Greenwood Publishing Co, 1972); Alfred W. Crosby, *Ecological Imperialism: The Biological Expansion of Europe, 900-1900* (Cambridge: Cambridge University Press, 1986).

14 Brett M. Bennett, “Epilogue: A Global History of Species Introduction and Invasion: Reconciling Historical and Ecological Paradigms,” in *Environments of Empire: Networks and Agents of Ecological Change*, ed. Ulrike Kirchberger and Brett M. Bennett (Chapel Hill: University of North Carolina Press, 2020), 224-245, 224.

15 Ibid., 225.

16 Stefan Helmreich, “How Scientists Think; About ‘Natives,’ for Example. A Problem of Taxonomy among Biologists of Alien Species in Hawaii,” *Journal of the Royal Anthropological Institute* 11 (2005): 107-128.

17 Daniel Simberloff, “Confronting Introduced Species: A Form of Xenophobia?” *Biological Invasions* 5 (2003): 179–192; Jonah H. Peretti, “Nativism and Nature: Rethinking Biological Invasion,” *Environmental Values* 7 (1998): 183–192; Brendon M.H. Larson, “Reweaving Narratives about Humans and Invasive Species,” *Études Rurales* 185 (2010): 25–38; Ned Hettinger, “Exotic Species, Naturalisation, and Biological Nativism,” *Environmental Values* 10 (2001): 193–224; Mark Woods and Paul Veatch Moriarty, “Strangers in a Strange Land: The Problem of Exotic Species,” *Environmental Values* 10 (2001): 163–191.

18 Bennett, “Epilogue,” 225.

19 See for example, Crystal Fortwangler, “A Place for the Donkey: Natives and Aliens in the US Virgin Islands,” *Landscape Research* 34 (2009): 205-222; Priscilla M. Ehi, Katie L. Kamelamela, Kyle Whyte, Krushil Watene, Nicholas Reo, “Contribution of Indigenous Peoples’ Understandings and Relational Frameworks to Invasive Alien Species management,” *People and Nature* 5 (2023): 1403-1414; David. S. Trigger, “Indigeneity, Ferality, and What ‘Belongs’ in the Australian Bush: Aboriginal Responses to ‘Introduced’ Animals and Plants in a Settler-Descendant Society,” *Journal of the Royal Anthropological Institute* 14 (2008): 628-646; Amelia Moore, “The Aquatic Invaders: Marine Management Figuring Fishermen, Fisheries, and Lionfish in The Bahamas,” *Cultural Anthropology* 27 (2012): 667-688; Jacob Tropp, “Locust Invasions and Tensions over Environmental and Bodily Health in the Colonial Transkei,” in *Indigenous Knowledge and the Environment in Africa and North America*, ed. David Gordon and Shepard Krech (Athens: Ohio University Press, 2012), 113–128.

20 Lance van Sittert, “‘Our Irrepressible Fellow-Colonist’: The Biological Invasion of Prickly Pear (Opuntia Ficus-Indica) in the Eastern Cape c.1890–c.1910,” *Journal of Historical Geography* 28 (2002): 397-419, 398.

21 Ibid.

22 Eben Kirksey, *Emergent Ecologies* (Durham: Duke University Press, 2015), 100.

23 Peter A. Coates, *American Perceptions of Immigrant and Invasive Species: Strangers on the Land* (Berkeley: University of California Press, 2006).

24 Shinozuka, *Biotic Borders*.

25 Ibid., 6. For other relationships between invasives and anti-Asian sentiments see for example, Derek Lee Nelson, “The Ravages of Teredo: The Rise and Fall of Shipworm in US History, 1860–1940,” *Environmental History* 21 (2016): 117. For related sentiments in Australia see Gilbert Caluya, “Fragments for a Postcolonial Critique of the Anthropocene: Invasion Biology and Environmental Security,” in *Rethinking Invasion Ecologies from the Environmental Humanities*, ed. Jodi Frawley and Iain McCalman (London: Routledge, 2014), 31–44.

26 Jodi Frawley and Iain McCalman, “Invasion Ecologies. The Nature/Culture Challenge,” in *Rethinking Invasion Ecologies from the Environmental Humanities*, 3–14

27 Emily Wanderer, *The Life of a Pest: An Ethnography of Biological Invasion in Mexico* (Berkeley: University of California Press, 2020), 45.

28 Ibid., 46.

29 Laura A. Ogden, “The Beaver Diaspora: A Thought Experiment,” *Environmental Humanities* 10 (2018): 63–85, 78.

30 Matthew Holmes, “A Plague of Weasels and Ticks: Animal Introduction, Ecological Disaster, and the Balance of Nature in Jamaica, 1870–1900,” *British Journal for the History of Science* 56 (2023): 391-407.

31 Ibid.

32 Ibid., 407. For discussions of problems of multispecies history and ethnography in colonial contexts in general, see Jonathan Saha, *Colonizing Animals* (Cambridge: Cambridge University Press, 2021), Introduction; Lesley Green, “Thinking Decoloniality with Perlemoen,” *Catalyst* 3 (2017): 25–30; Lesley Green, *Rock | Water | Life: Ecology and Humanities for a Decolonial South Africa* (Durham: Duke University Press, 2020).

33 Jackson R. Perry, “‘Conquered by the Sparrows’: Avian Invasions in French North Africa, circa 1871–1920,” *Environmental History* 25 (2020): 318.

34 Ibid., 316.

35 Frederick FitzSimons, 1920, quoted in Jules Skotnes-Brown, *Segregated Species: Pests, Knowledge, and Boundaries in South Africa, 1910-1948* (Baltimore: Johns Hopkins University Press, 2024), 120. See also, Harriet Ritvo, “Back Story: Migration, Assimilation and Invasion in the Nineteenth Century,” in *Rethinking Invasion Ecologies from the Environmental Humanities*, 17–30; van Sittert, ‘“Our Irrepressible Fellow-Colonist.”

36 Skotnes-Brown, *Segregated Species,* 143.

37 Haripriya Rangan and Christian Kull, “The Indian Ocean and the Making of Outback Australia: An Ecocultural Odyssey,” in *Indian Ocean Studies: Cultural, Social, and Political Perspectives*, ed. Shanti Moorthy and Ashraf Jamal (London: Routledge, 2010), 45–72.

38 Kezia Barker and Robert A. Francis, ed., *Routledge Handbook of Biosecurity and Invasive Species* (London: Routledge, 2021); Andrew Dobson, Kezia Barker, and Sarah L. Taylor, *Biosecurity: The Socio-politics of Invasive Species and Infectious Diseases* (Abingdon: Routledge, 2013); Giuseppe Mazza and Elena Tricarico, ed., *Invasive Species and Human Health* (Boston: CABI, 2018); Alfred W. Crosby, “Virgin Soil Epidemics as a Factor in the Aboriginal Depopulation in America,” *William and Mary Quarterly* 33 (1976): 289-299; David S. Jones, “Virgin Soils Revisited,” *William and Mary Quarterly* 60 (2003): 703-42; Paul Kelton, *Cherokee Medicine, Colonial Germs. An Indigenous Nation’s Fight Against Smallpox, 1518-1824* (Norman, OK: University of Oklahoma Press, 2015); Nathan Nunn and Nancy Qian, “The Columbian Exchange: A History of Disease, Food, and Ideas,” *Journal of Economic Perspectives* 24 (2010): 163–188; Noble David Cook, *Born to Die: Disease and New World Conquest, 1492-1650* (Cambridge: Cambridge University Press, 1998).

39 Afrânio Peixoto, 1941, quoted in Gabriel Lopes, “Anopheles Gambiae in Brazil: The Background to a ‘Silent Spread,’ 1930-1932,” *História, Ciências, Saúde-Manguinhos* 26 (2019), https://doi.org/10.1590/S0104-59702019000300006. See also Karen Sayer’s discussion of plague and rats as invasive in south England. Karen Sayer, “Vermin Landscapes: Suffolk, England, Shaped by Plague, Rat and Flea (1906-1920),” in *Framing Animals as Epidemic Villains*, ed. Christos Lynteris (Cham: Palgrave Macmillan, 2019), 27–64.

40 Charles S. Elton, “Plague and the Regulation of Numbers in Wild Mammals,” *Journal of Hygiene* 24 (1925): 138-163; Charles S. Elton, *Animal Ecology* (New York: Macmillan, 1925); Mark Honigsbaum, “‘Tipping the Balance’: Karl Friedrich Meyer, Latent Infections, and the Birth of Modern Ideas of Disease Ecology,” *Journal of the History of Biology* 49 (2016): 261-309. For an analysis of how settler colonies were key sites for the development of disease ecology as a science, see Warwick Anderson, “Postcolonial Ecologies of Parasite and Host: Making Parasitism Cosmopolitan,” *Journal of the History of Biology* 49 (2016): 241–259.

41 Myron J. Echenberg, *Plague Ports: The Global Urban Impact of Bubonic Plague, 1894–1901* (New York: New York University Press, 2007).

42 Honigsbaum, “’Tipping the Balance’”; Susan D. Jones and Anna A. Amramina, “Entangled Histories of Plague Ecology in Russia and the USSR,” *History and Philosophy of the Life Sciences* 40 (2018): https://doi.org/10.1007/s40656-018-0220-3.

43 Jules Skotnes-Brown, “Scurrying Seafarers: Shipboard Rats, Plague, and the Land/Sea Border,” *Journal of Global History* 18 (2023): 108–130; Lukas Engelmann and Christos Lynteris, *Sulphuric Utopias: A History of Maritime Fumigation* (Cambridge, MA: MIT Press, 2020).

44 On “epidemic villain” see Christos Lynteris, ed., *Framing Animals as Epidemic Villains*.

